# Beyond distance: integrating economic burden into large-scale primary healthcare accessibility analysis

**DOI:** 10.1186/s41256-025-00451-9

**Published:** 2025-10-27

**Authors:** Bin Zhu, Liutong Chen, Yifei He, Ning Zhang, Hao Xue, Samir Bhatt, Minghui Ren, Ying Mao

**Affiliations:** 1 School of Public Health and Emergency Management, Southern University of Science and Technology, Shenzhen, 518055 China; 2 State Key Laboratory of Earth Surface Processes and Resource Ecology, Faculty of Geographical Science, Beijing Normal University, Beijing, 100875 China; 3 Vanke School of Public Health, Tsinghua University, Beijing, 100084 China; 4 Stanford Center On China’s Economy and Institutions, Freeman Spogli Institute for International Studies, Stanford University, Stanford, USA; 5 MRC Centre for Global Infectious Disease Analysis and the Abdul Latif Jameel Institute for Disease and Emergency Analytics, School of Public Health, Imperial College, London, UK; 6 Section of Epidemiology, Department of Public Health, University of Copenhagen, Copenhagen, Denmark; 7 Department of Global Health, School of Public Health, Beijing, 100191 China; 8 School of Public Policy and Administration, Xi’an Jiaotong University, Xi’an, 710049 China

**Keywords:** Primary healthcare, PHC accessibility, Travel costs, Economic burden, Geospatial analysis, Chinese mainland

## Abstract

**Background:**

Access to primary healthcare (PHC) services is a critical determinant of population health outcomes and a key indicator of health system performance. However, comprehensive methods for batch assessment of PHC accessibility and the associated economic burden are relatively scarce. This gap is particularly evident in developing countries, where accessibility challenges are often more pronounced.

**Methods:**

We developed an integrated assessment methodology for the large-scale calculation of travel costs to access PHC services. This methodology combines high-resolution friction surface mapping, the construction of a comprehensive PHC facility spatial dataset, and the application of least-cost path algorithms. The batch processing capability of the methodology ensures efficient computation while maintaining high spatial resolution, enabling a detailed evaluation of PHC accessibility and its economic burden across diverse geographical contexts.

**Results:**

Our analysis revealed that 88.70% of Chinese mainland’s population could access PHC facilities within an hour, indicating relatively good overall accessibility to primary healthcare services. However, significant regional disparities in accessibility and associated economic burdens were observed. While urban areas, particularly in eastern and coastal regions, generally exhibit high levels of accessibility, rural and remote areas, especially in the western and northwestern regions, face substantial challenges in reaching PHC facilities. The total economic burden associated with travel to PHC facilities in Chinese mainland is estimated at approximately 38.29 billion CNY annually. The southern, northern, and northwestern regions accounted for 62.91%, 21.72%, and 14.94% of the total burden respectively, with northwestern provinces facing a disproportionately high economic burden relative to their GDP.

**Conclusions:**

Our methodology supports future large-scale, high-resolution accessibility analyses that can guide interventions aimed at addressing healthcare disparities and improving equity. The empirical application of this methodology in Chinese mainland revealed significant disparities in PHC access across the country and quantified the substantial economic costs associated with these disparities.

**Supplementary Information:**

The online version contains supplementary material available at 10.1186/s41256-025-00451-9.

## Introduction

The primary healthcare (PHC) institutions are designed to meet the fundamental health needs of individuals and communities with an emphasis on universal accessibility. As outlined in the 1978 Declaration of Alma-Ata [1], PHC aims to provide a first point of contact for healthcare within the community, focusing on accessible, preventive, promotive, curative, and rehabilitative services. Forty years later, the Declaration of Astana further prioritizes equity and emphasizes the importance of community participation in managing and improving health, with a goal of reducing health disparities and promoting overall well-being at the grassroots level.

PHC accessibility is a critical determinant of population health outcomes and a key indicator of health system performance [2]. The global distribution of PHC resources exhibits significant disparities, with rural and remote areas often facing prolonged travel times and increased costs when accessing medical services [3]. This inequity is exacerbated by inadequate transportation infrastructure and the absence of motorized transport, disproportionately affecting lower-income groups [4]. Consequently, individuals encountering extended travel times to PHC facilities are less inclined to seek medical attention, when necessary, potentially leading to elevated rates of mortality and morbidity from treatable conditions [5]. Recognizing the importance of PHC accessibility, the United Nations Sustainable Development Goals (SDGs) explicitly address this issue in Target 3.8, which aims to achieve universal health coverage, including financial risk protection and access to quality essential PHC services [6]. In this context, the accurate measurement and analysis of PHC accessibility, particularly in terms of travel costs, have become increasingly crucial for informing health policy and resource allocation decisions.The conceptualization and measurement of healthcare accessibility have evolved significantly over the past few decades. Traditional approaches often relied on simplistic measures such as provider-to-population ratios or Euclidean distances [7]. However, these methods failed to capture the complexities of real-world travel and spatial distribution of both healthcare facilities and populations. More sophisticated methods emerged with the advent of Geographic Information Systems (GIS), enabling researchers to incorporate spatial data and conduct more nuanced analyses of healthcare accessibility [8]. For example, the two-step floating catchment area method and its variants have been widely adopted to assess spatial accessibility to healthcare services, considering both supply and demand factors [9, 10]. These methods have provided valuable insights into healthcare accessibility patterns but often focus on localized areas or specific types of healthcare services.Recent advancements in geospatial technologies and data availability have paved the way for more comprehensive assessments of healthcare accessibility. Studies have increasingly utilized road network data from sources such as OpenStreetMap to calculate more accurate travel times [4]. These approaches assign different speeds to various road types and consider factors such as terrain and land use when estimating travel times. For instance, Hierink et al. [11] developed a high-resolution map of travel time to healthcare facilities in sub-Saharan Africa, integrating multiple data sources to account for various modes of transportation and geographical barriers. Similarly, Munoz and Källestål [12] employed GIS techniques to analyze geographical accessibility to health facilities in rural Tanzania, highlighting the potential of spatial analysis in identifying underserved areas. Despite these advancements, challenges remain in developing methodologies that can efficiently calculate travel costs for healthcare access across large and diverse geographical areas, particularly in a batch processing.The literature also reveals a growing recognition of the need to consider not only physical accessibility but also the associated economic burdens of healthcare access. Travel costs, including both direct expenses and the opportunity costs of travel time, can significantly impact healthcare utilization patterns and outcomes [13]. However, comprehensive assessments of these economic burdens, particularly on large scales, remain limited. Futhermore, existing studies often focus on single-way travel costs, neglecting the round-trip nature of healthcare visits and potentially underestimating the true burden of healthcare access. For example, Zhao et al. [14] analyzed hospital accessibility based on residential location but focused only on single-way travel. Similarly, Kotavaara et al. [15] examined travel chains to health services without incorporating round-trip dynamics. Wang et al. [16] assessed PHC accessibility in Sichuan, China, yet did not account for the cumulative burden of round-trip travel. This gap underscores the need for holistic approaches that can capture both the spatial and economic dimensions of healthcare accessibility across diverse geographical contexts.

We aim to addresses these gaps by developing a comprehensive methodology for the batch calculation of travel costs to access PHC services. The proposed approach integrates multiple global and national databases within an advanced geospatial modelling framework, providing a more nuanced and accurate representation of travel impedance across diverse geographical contexts. Furthermore, by applying this approach to Chinese mainland, we gain insights into the spatial distribution of PHC accessibility and the associated economic burden in one of the world’s most populous countries. This will serve as a reference for the allocation of PHC resources around the world, especially in developing countries.

## Methodology

### Conceptual framework for batch travel cost calculation

The proposed framework for the batch calculating travel costs to access PHC services addresses the need for efficient, large-scale assessments of PHC accessibility. It integrates multiple geospatial datasets and advanced analytical techniques, overcoming limitations of existing approaches that often rely on simplistic measures such as provider-to-population ratios or Euclidean distances [17]. By incorporating real-world travel complexities and spatial distributions of PHC facilities and populations, this framework enables comprehensive analysis across diverse geographical contexts.

The conceptual foundation of our framework rests on four key points: (1) comprehensive spatial representation, integrating diverse geospatial data sources to create a high-resolution friction surface map [18, 19]; (2) accurate facility localization, ensuring precise geocoding and spatial representation of PHC facilities [20]; (3) advanced travel time algorithms, employing a least-cost path algorithm to calculate the most efficient routes between population centers and PHC facilities [21]; and (4) economic burden quantification, assessing the economic burden associated with PHC access [22].

A key innovation of the proposed framework is its batch processing capability, which is designed for efficient large-scale computations. This feature enables the analysis of vast geographical areas while maintaining high spatial resolution, addressing a significant gap in existing methodologies that often struggle to balance computational efficiency with geographical detail [23]. By integrating these components sequentially, our framework facilitates the simultaneous processing of multiple geographical units, thereby enhancing the efficiency and comprehensiveness of PHC accessibility analyses.

### Study design

**Fig. 1:**
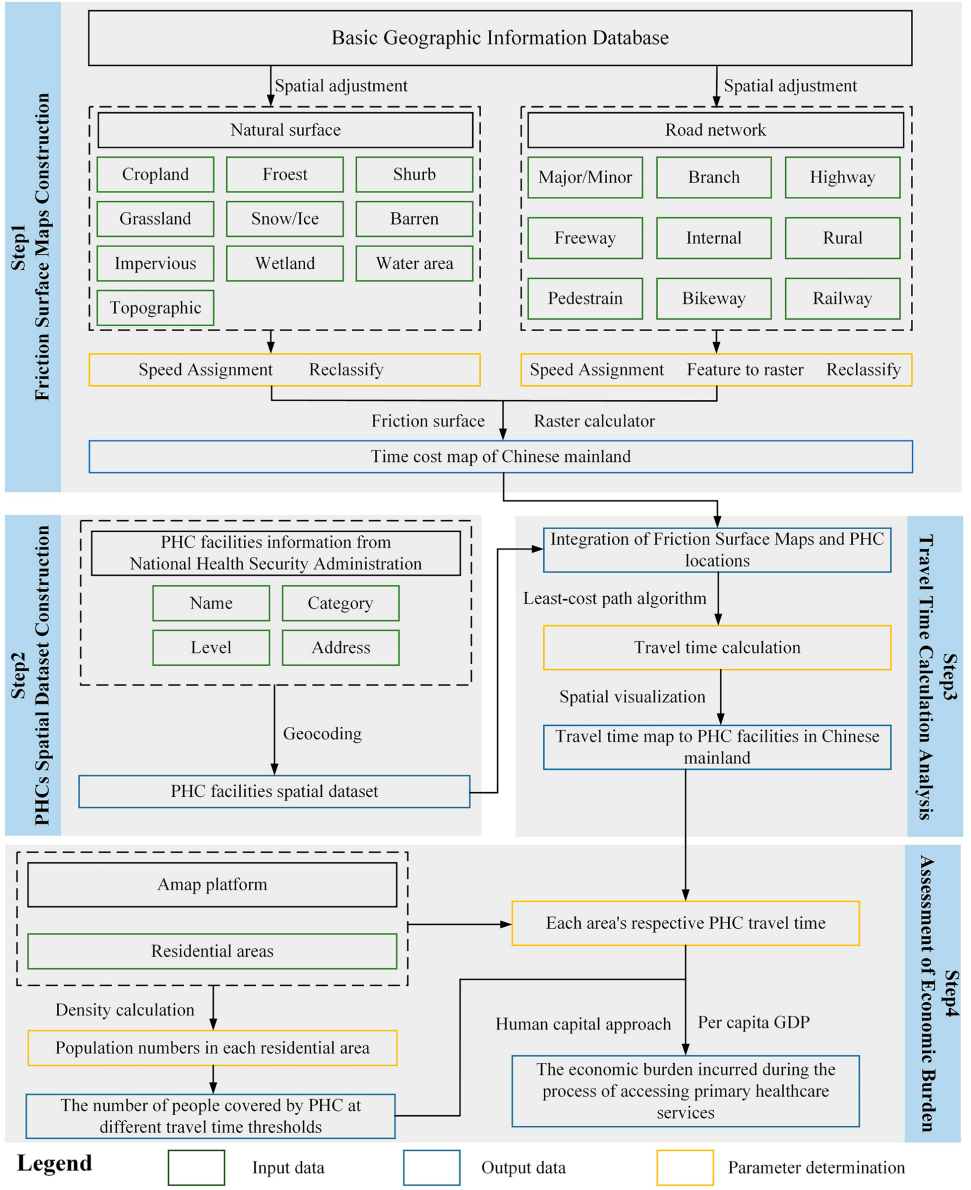
Flowchart for calculating economic burden to PHC in Chinese mainland. Notes: Based on the spatial adjustment of the multi-source basic geographic information database, the speed settings are set for different surface features, finally combining the raster calculation and the least cost distance algorithm to form the PHC travel time map of Chinese mainland

Our study design comprises a four-step sequential approach, enabling efficient large-scale assessment of PHC accessibility while maintaining high spatial resolution (Fig. 1). Each step builds upon the previous step, creating a comprehensive methodology for the batch calculation of travel costs to access PHC services.

#### Friction surface map generation

A high-resolution friction surface map was generated using raster calculation methods, integrating road networks, terrain data, and land use information. The time cost for traversing each cell is defined as:

1 $$\text{cost}=1/v$$

where *v* denotes the travel speed assigned to each cell based on its characteristics. The spatial resolution was set at 1 × 1 km to balance computational efficiency and spatial detail.

Travel speed (*V*) is determined by road type, land use, and topography. To account for topographic effects, we employ the following modified Tobler’s bike function [24]:

2 $$V={V}_{0}*exp \left(-k\left|s\right|\right)$$

where ${V}_{0}$ is the base speed on flat terrain, $k$ is the terrain coefficient, and $s$ is the slope. This function models the impact of terrain on travel speeds, particularly in areas with significant elevation changes.

The data sources used in our analysis included open-source road network databases, global land cover datasets, and digital elevation models, which were processed and integrated using GIS software to create a final friction surface map.

#### PHC facility spatial dataset construction

A comprehensive spatial dataset of PHC facilities was constructed through the following steps: 1) Facility identification: PHC facilities were compiled from official databases and classified according to standardized categories. 2) Geocoding: we employing a multi-stage process including automated geocoding using multiple application programming interfaces (APIs), cross-referencing results, and manual verification of low-confidence cases. 3) Data cleaning and validation: Removing duplicates and validating locations using satellite imagery and street-view services. 4) Attribute assignment: assigning relevant attributes such as facility type, service level, and administrative unit. The resulting dataset provides a comprehensive representation of PHC facility distribution, which is crucial for subsequent travel time calculations.

#### Travel time calculation and mapping

This phase encompasses the comprehensive analysis of travel times to PHC facilities, employing the following methodology: 1) Integration of friction surface and facility data: the friction surface maps (Step 1) are integrated with the PHC facility spatial dataset (Step 2) through geospatial overlay techniques, enabling the association of each facility with local travel impedance characteristics. 2) Travel time computation: A least-cost path algorithm was implemented to calculate travel times from residential areas to the nearest PHC facilities. This algorithm incorporates heterogeneous time costs defined by the friction surface. The travel time *T* for a path is computed as:

3 $$T=\Sigma \left(1/{v}_{i}\right)$$

where ${v}_{i}$ denotes the travel speed for the *i*th path segment.

3) Accessibility analysis: accessibility analysis was conducted based on the computed travel times. Regions were categorized using predefined travel time thresholds (e.g., 15, 30, and 60 min), facilitating the identification of areas with varying levels of PHC accessibility. 4) Spatial visualization: the travel time analysis results were visualized using GIS software, generating isochrone maps that illustrate the spatial distribution of PHC accessibility. These visualizations highlight areas of significant travel burden, indicating potential intervention strategies.

#### Economic burden assessment

The economic burden associated with PHC access was assessed quantitatively using a human capital approach. This method considers the economic burden incurred by residents accessing PHC services. In line with the human capital approach and health economics principles, we define economic burden as burden arising from the time loss incurred by residents when accessing PHC services, including the economic costs of work time loss and travel time loss. It is important to note that the time burden calculation adopted in this study includes only the travel time to and from PHC facilities. Waiting and consultation times with doctors were not considered in this assessment because our focus was on the accessibility of PHC services in terms of travel costs. Total economic burden (EB) is calculated using the following equation:

4 $$\text{EB}={\Sigma }_{\text{m}=1}^{\text{M}}{\Sigma }_{\text{j}=1}^{\text{K}}\left({\text{R}}_{\text{mj}}*{\text{T}}_{\text{ij}}*\text{V}*{\text{P}}_{\text{m}}\right)/365$$

where *m* represents provincial administrative units, *M* represents the number of provincial administrative units in this study, and *M* = 31, *j* represents residential areas, *K* is the total number of residential areas obtained from the Gaode map platform, ${R}_{mj}$ is the population of the residential area, ${T}_{ij}$ is the lost workday fraction, *V* is the average number of PHC visits per year, and ${P}_{m}$ is the per capita GDP.

The lost workday fraction ${T}_{ij}$ is derived from the travel time calculated as described in Sect. "Travel time calculation and mapping", which is converted to a proportion of a standard workday (typically 8 h). The annual visit rate *V* is obtained from national health statistics, potentially stratified by age groups or socioeconomic factors where data permits. This formulation allows for a spatially explicit assessment of the economic burden, accounting for variations in population distribution, PHC accessibility, utilization patterns, and economic productivity across different administrative units. Therefore, our results provide a monetary quantification of accessibility challenges and offer valuable insights for policy makers and healthcare planners.

The proposed model’s scalability enables analysis at various administrative levels from local to national. Statistical techniques such as Monte Carlo simulation has the potential to account for uncertainties in the input parameters, providing confidence intervals for the estimated economic burden. This economic burden assessment, in conjunction with the accessibility analysis from the previous step, provides a comprehensive evaluation of PHC access disparities and their economic implications, forming a robust basis for evidence-based policy formulation and resource allocation decisions.

### Software

The entire data analysis process was implemented using Python 3.10 for computational tasks and ArcGIS 10.4.1 for spatial operations and visualization, enabling the efficient batch processing of large datasets.

## Empirical application in Chinese Mainland

### Study area and data sources

#### Overview of the Chinese healthcare system

The Chinese healthcare system has undergone significant reforms in recent decades, to provide universal health coverage and improve PHC accessibility. The system is characterized by a three-tiered structure of healthcare facilities consisting of primary, secondary, and tertiary facilities. PHC facilities, including community health centers in urban areas and township health centers in rural areas, form the foundation of the system and are the focus of this study. These facilities are designed to provide basic medical services, disease prevention, and health promotion to local communities. In Chinese mainland, PHC institutions provided 55% of outpatient care and 18% of inpatient care in 2016 [25].

Despite substantial progress, disparities in healthcare accessibility persist, particularly between urban and rural areas and across different regions of Chinese mainland. These disparities are influenced by factors such as uneven economic development, varying population densities, and geographical constraints [26]. Understanding and quantifying these disparities are crucial for informed policy-making and resource allocation in the world’s most populous countries.

#### Data collection and preparation

To implement the proposed methodology for assessing PHC accessibility and the economic burden in Chinese mainland, a comprehensive dataset was compiled from multiple sources. The data collection process focused on four key areas: road networks and transportation infrastructure, land use and topography, PHC facility locations, and socioeconomic indicators. Table 1 provides an overview of the datasets used in this study, including their sources, spatial resolutions, and temporal coverage.

**Table 1:** Datasets used in this study

Purpose	Database	Type	Speed (km/h)	Resolution	Periods	Source
Travel time calculation	Road network	Major roads	60	Chinese mainland	2022	Open Street Map (https://www.openstreetmap.org/relation/3464353)
		Minor roads	50			
		Branch roads	40			
		Freeway roads	100			
		Highway roads	120			
		Internal roads	20			
		Rural road	30			
		Pedestrian	3.6			
		Bikeway	15			
	Land use	Cropland	2.5	30 m	2021	Yang et al. (2021)
		Forest	4.05			
		Shrub	3.6			
		Grassland	4.86			
		Snow/Ice	1.62			
		Barren	3			
		Impervious	5			
		Wetland	2			
	Railway	–	210	Chinese mainland	2022	Open Street Map (OSM) (https://www.openstreetmap.org/relation/3464353)
	Water area	–	20	Chinese mainland	2022	National Geomatics Center of China (https://www.webmap.cn/commres.do?method=result100W)
	Topographic	–	–	15" (approximately 500 m)	2022	General Bathymetric Chart of the Oceans (GEBCO) (https://www.gebco.net/data_and_products/gridded_bathymetry_data/)
Economic burden calculation	Residential areas	–	–	Chinese mainland	2022	Gaode Map (https://www.amap.com/)
	Administrative boundary	–	–	Chinese mainland	2019	Ministry of Natural Resources (http://bzdt.ch.mnr.gov.cn/)
	Per capita GDP	–	–	Chinese mainland	2021	National Bureau of Statistics (https://data.cnki.net/)
	Number of visit to PHC	–	–	Chinese mainland	2021	National Health Commission of China (https://www.stats.gov.cn/sj/ndsj/2021/indexch.htm)

The PHC facility locations were obtained from the National Health Security Administration of China (https://fuwu.nhsa.gov.cn). This database provides comprehensive information about medical institutions across Chinese mainland, including facility names, categories, levels, and detailed addresses. The dataset was processed to focus specifically on PHC facilities, in line with the objectives. The road network and railway data were obtained from OpenStreetMap, which provides detailed information on road types, including highways, major roads, minor roads, and rural paths. These data are crucial for generating a friction surface map. Land use data were sourced from Yang et al. [27], who provided a high-resolution (30 m) land cover classification for Chinese mainland. Topographic data were acquired from the General Bathymetric Chart of Oceans (GEBCO), which provides elevation information at a resolution of approximately 500 m. Socioeconomic data, including population distribution, GDP, and PHC utilization statistics, were collected from multiple sources. Population data at the residential area level were collected from the Gaode Map platform, which provides detailed information on population distributions. Administrative boundary data were obtained from the Ministry of Natural Resources of China, allowing analysis at various administrative levels. Economic data, including per-capita GDP, were sourced from the National Bureau of Statistics of China.

The integration of these diverse datasets required careful preprocessing and quality control. Geocoding of the PHC facilities was performed using the API service of the Amap platform, with manual verification of a subset of locations to ensure accuracy. Duplicate entries were removed, and spatial analysis methods were employed to standardize the data across different sources and resolutions. This comprehensive dataset forms the foundation for the subsequent application of the proposed methodology, enabling a detailed analysis of PHC accessibility and the associated economic burdens across Chinese mainland.

### Implementation of the proposed methodology

#### Friction surface map generation for Chinese mainland

A friction surface map of Chinese mainland was generated using a combination of national and global datasets. To account the varied topography of Chinese mainland from the Tibetan Plateau to the eastern coastal plains, a region-specific modification of the terrain coefficient in Tobler’s bike function was implemented. This modification was performed based on empirical studies conducted on different Chinese landscapes [28]. Therefore, the topographic correction factor in Eq. (2) was carefully calibrated with *k* values adjusted based on local studies of travel patterns in these regions.

5 $$k = 6e^{{ - 3.5\tan \left( {0.01745 \times {\text{slopeangle}}} \right) + 0.05}} /5.0$$

#### Construction of the PHC facilities dataset

The construction of a PHC facility dataset for Chinese mainland requires the navigation of a complex healthcare system. Data from the National Health Security Administration were supplemented with provincial health department records to ensure comprehensive coverage, particularly for rural health stations, which are often underrepresented in national databases. The geocoding process faces challenges as a result of inconsistent address formats across different regions of Chinese mainland. A multistep approach was developed using a combination of Baidu Maps and the Amap API, with manual verification of facilities in remote areas. This process achieved a geocoding success rate of 98.7%, which is significantly higher than that of previous national-scale studies.

#### Travel time calculation and accessibility analysis

Travel time calculations in Chinese mainland must consider the country’s diverse transportation modes. In urban areas, our calculation of healthcare access incorporated data on the public transportation systems in major cities. In contrast, for rural areas, informal transportation networks, which are typically used for local travel between villages and fields, were considered. These networks, which are outside the formal national highway system, are primarily designed to support agricultural activities and rural mobility. They play an important role in accessing healthcare in areas with limited formal transportation infrastructure.

#### Economic burden assessment in the Chinese context

Our economic burden assessment is tailored to China’s unique socioeconomic landscape. Our analysis was conducted at the provincial level, using high-resolution residential data from the Gaode map platform. Province-specific per capita GDP data from the National Bureau of Statistics were used to reflect regional economic disparities. The lost workday fraction calculation considered urban–rural differences in the number of PHC visits, and travel time estimations incorporated China’s diverse transportation modes, including public transit in urban areas and informal networks in rural regions. The average number of PHC visits per year was obtained from the 2021 National Health Statistical Book, with 3.01 annual visits to PHC facilities per year (1.43 to community/township health centers and 1.59 to clinics). These adaptations allowed for a nuanced assessment of the economic burden associated with PHC accessibility, capturing the complexities of China’s healthcare system and socioeconomic variations across its vast territory. The results provide insights into the regional disparities and economic implications of PHC access challenges specific to Chinese mainland.

### Friction surface map

#### Nationwide friction surface map characteristics

**Fig. 2:**
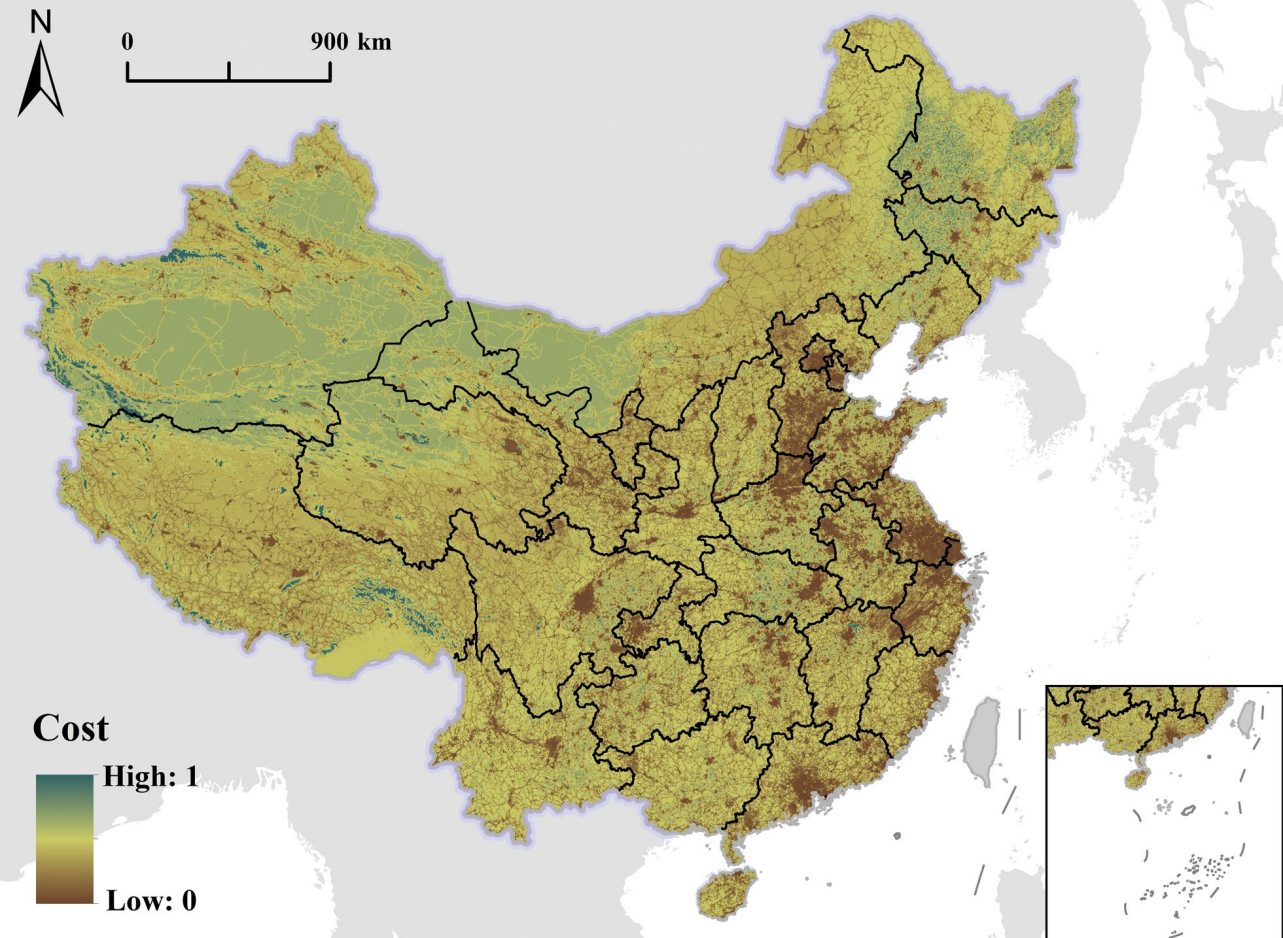
Time cost map of Chinese mainland in 2022

Based on the friction surface maps constructed as described in Sect. "Friction surface map generation for Chinese mainland", a comprehensive time cost map for PHC access in Chinese mainland was developed for 2022 (Fig. 2). This map illustrates the spatial distribution pattern of travel time costs across the country and provides a nuanced view of PHC accessibility on a national scale.

The nationwide friction surface map reveals a clear east-to-west gradient in travel impedance. Eastern China, particularly the coastal regions, exhibit lower friction values, indicating better accessibility to PHC facilities. In contrast, Western China exhibits significantly higher friction values. Urban centers across the country are characterized by localized areas of low friction, appearing as “islands” of high accessibility amid areas of higher travel impedance. This pattern is particularly pronounced in provincial capitals and major cities, highlighting the concentration of PHC resources in urban areas.

#### Regional variations in travel impedance

The friction surface map reveals substantial regional variations in travel impedance across Chinese mainland’s five major geographical divisions. (1) North China: this region, including Beijing, Tianjin, and parts of Inner Mongolia, exhibits moderate to low friction values. Urban areas, particularly Beijing and Tianjin, exhibit very low travel impedances. However, parts of Inner Mongolia exhibit higher friction values, indicating challenges in PHC access in remote areas. (2) Northeast China: Liaoning, Jilin, and Heilongjiang provinces present a mixed pattern. Urban centers exhibit low friction values but are surrounded by vast areas of higher impedance. This trend reflects the region’s historical industrial base in contrast to its more sparsely populated rural areas. (3) East China: this region, including Shanghai, Jiangsu, and Zhejiang, consistently exhibits the lowest friction values nationwide. The Yangtze River Delta area stands out as a zone with exceptionally low travel impedance, indicating excellent PHC accessibility. (4) South and Central China: this region encompasses provinces such as Guangdong, Hunan, and Henan, and exhibits a gradient of friction. The Pearl River Delta in Guangdong exhibits very low impedance, similar to that in East China. However, the friction values increase in the inland areas of this region, particularly in the mountainous parts of Hunan and Guangxi. (5) Southwest and Northwest China: these regions, including provinces such as Sichuan, Tibet, Xinjiang, and Qinghai, exhibit the highest friction values nationwide. The Tibetan Plateau and vast desert regions of Xinjiang exhibit extreme travel impedance as a result of challenging terrain and sparse population distributions. Major cities in these regions, including Chengdu and Xi’an, appear as isolated areas of low friction amidst generally high-impedance areas.

### Travel time and costs

#### Spatial distribution of travel times to PHC facilities

**Fig. 3:**
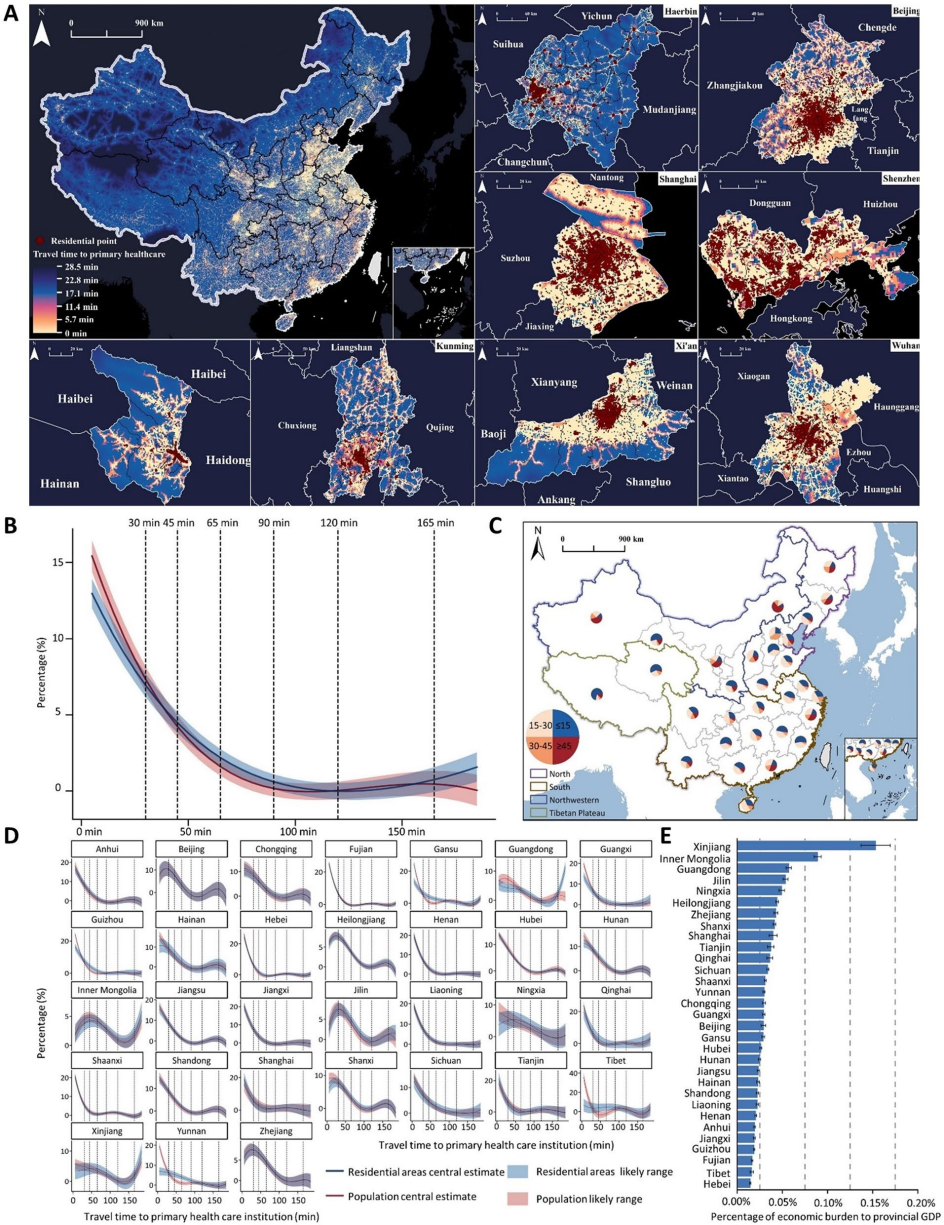
Travel time and costs to PHC facilities in Chinese mainland here. Notes: Panel A represents the travel time to PHC facilities in Chinese mainland, with representative cities from eight geographic directions selected to present their internal travel times to PHC facilities. Panel B describes the national proportion of population and geographical areas with differentiating access to PHC facilities. Panel C illustrates the proportions of the population with access to PHC at different time thresholds. Panel D shows the provincial proportion of the population and geographical areas with differentiating access to PHC facilities. Panel E displays the percentage of economic burden for seeking PHC to provincial GDP. (Map Approval Number: GS(2023)2767 Supervised By the Ministry of Natural Resources of the People’s Republic of China.)

The application of our methodology resulted in a high-resolution map quantifying the travel time to PHC facilities across Chinese mainland at a spatial resolution of approximately 1 × 1 km (Fig. 3A). This “Travel Time Map of Primary Healthcare Facilities in Chinese Mainland” provides a comprehensive understanding of residents’ accessibility to PHC services from national to intra-urban levels. The map reveals significant spatial disparities inaccessibility of PHCs. Urban areas, particularly in Eastern China, exhibit consistently short travel times, often within 15 min. In contrast, rural and remote areas, especially in the western regions, exhibit substantially longer travel times, frequently exceeding 60 min. This pattern aligns with the friction surface map characteristics discussed in Sect. "Friction surface map".

To illustrate intra-urban variations, we selected representative cities from eight geographic directions: Harbin (northeast), Beijing (north), Shanghai (east), Shenzhen (southeast), Wuhan (central), Xi’an (northwest), Kunming (southwest), and Xining (west). These city-level analyses reveal nuanced patterns of PHC accessibility within urban areas, highlighting the disparities between city centers and peripheral regions.

#### Population coverage analysis

Our analysis indicates that 88.70% of Chinese mainland’s population can access PHC facilities within an hour (Fig. 3B). However, this figure masks the significant regional variations. Approximately 70.93% of the population and 62.01% of the residential areas have access to PHC services within 30 min. Furthermore, approximately 41.58% of the total population and 35.51% of residential areas can access these services within 15 min (Fig. 3C).

Figure 3D presents the provincial breakdown of population and geographical area coverage at different time thresholds. Eastern provinces generally exhibit higher percentages of the population covered within shorter time frames than western provinces, reflecting the east–west divide in PHC accessibility.

Approximately 11.3% of the population requires more than 60 min to access PHC services, with these individuals primarily concentrated in the northwest, southwest, and Tibetan Plateau regions of China. The analysis results indicate a significant positive correlation between road network density and travel time (r = 0.50, *p* < 0.05), suggesting that underdeveloped transportation networks are the primary constraints on healthcare accessibility in remote areas. For example, in regions such as Tibet and Qinghai, the combination of complex terrain and low road network density results in significantly longer average travel times to healthcare facilities than the national average. Additionally, population density exhibits a weak negative correlation with travel time (r =  − 0.23, *p* < 0.05), indicating that in sparsely populated areas such as southern Xinjiang, the sparse distribution of healthcare resources further increases travel time. Additionally, the results indicate that in highly populated urban areas such as the central districts of Beijing or Shanghai, despite the concentration of healthcare resources, traffic congestion may increase the time required for residents to access healthcare services.

#### Economic burden estimates across regions

The total economic burden associated with travel to PHC facilities in Chinese mainland is estimated at approximately 38.29 billion CNY (27.35 CNY per capita) annually. According to the “2023 China Health and Wellness Development Statistical Bulletin,” Chinese mainland’s total healthcare expenditure in 2023 was approximately 9.06 trillion CNY. This figure indicates that the economic waste caused by accessing PHC facilities accounts for as much as 0.42% of the total healthcare expenditure. A study in India [29] indicated that the government would need to pay approximately 3.57 USD (26 CNY) per capita at the PHC level under a capitation-based provider payment model for comprehensive PHC services. This result highlights the relatively high economic burden faced by Chinese people when accessing PHC services.

Additionally, this figure highlights the significant hidden costs of PHC access disparities. Our regional analysis revealed that the economic burden is not evenly distributed across Chinese mainland’s geographical divisions. The southern, northern, and northwestern regions account for 62.91%, 21.72%, and 14.94% of the total burden, respectively. However, when examining individual provinces, the economic burden as a percentage of provincial GDP is significantly higher in the northwestern provinces than in the southeastern provinces (Fig. 3E).

**Fig. 4:**
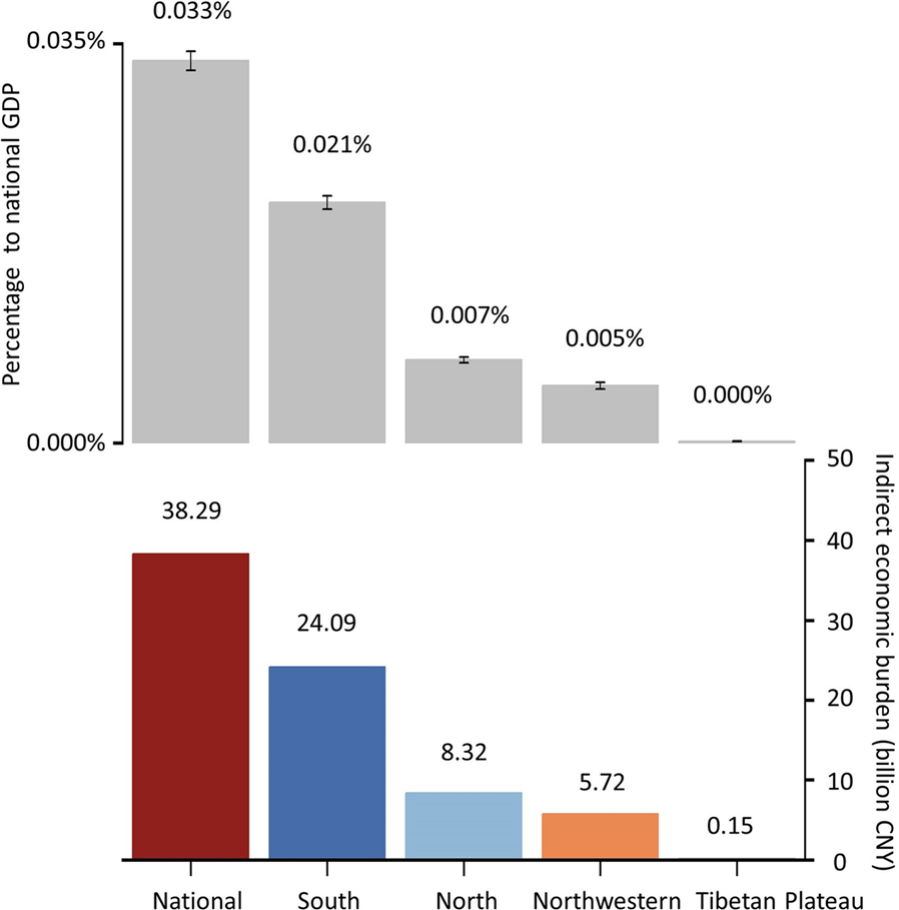
Economic burden incurred during the process of accessing PHC services in Chinese mainland

Figure 4 presents a detailed breakdown of the economic burden across Chinese mainland’s geographical units. South China faced the highest economic loss of approximately 24.09 billion CNY in 2021, reflecting the high population density and frequent PHC utilization in this economically developed region. The north, northwestern, and Tibetan Plateau regions faced economic losses of approximately 8.32 billion CNY, 5.72 billion CNY, and 0.15 billion CNY, respectively.

#### Technical validation

To ensure the reliability of our travel time estimates, we employed external validation using data from a reputable map service platform—a method frequently adopted in transportation and health accessibility studies. Specifically, we used the Route Planning API of Baidu Map (https://lbsyun.baidu.com/), a leading and authoritative navigation provider in China, as the reference benchmark.

To validate the estimated travel times generated by our methodology, we randomly selected four provinces across different regions of Chinese mainland: Zhejiang (eastern region), Hubei (central region), Ningxia (northwestern region), and Guizhou (southwestern region). For each province, origin–destination (OD) coordinate pairs were constructed by setting each 1 km grid cell as the origin (O) and its nearest healthcare facility as the destination (D). After removing invalid and anomalous records, we obtained a final validation dataset containing OD coordinates and corresponding travel time estimates. A sample of this dataset is presented in Table 2.

**Table 2:** A sample record of the validation datasets

Province	O_lon	O_lat	D_lon	D_lat	Our model (min)	Baidu API (min)
Zhejiang	119.78188	31.17694	119.73788	31.10942	17.92	24.75
Hubei	112.24855	31.56861	112.24414	31.55051	19.30	22.55
Ningxia	106.74021	39.38527	106.71830	39.26353	20.23	22.47
Guizhou	106.74855	27.82694	106.72847	27.81612	28.59	34.68

O_lon: Longitude of the origin; O_lat: Latitude of the origin; D_lon: Longitude of the destination; D_lat: Latitude of the destination; Our model (min): Estimated travel time in minutes calculated by our proposed model; Baidu API (min): Estimated travel time in minutes retrieved from the Baidu Map API

**Fig. 5:**
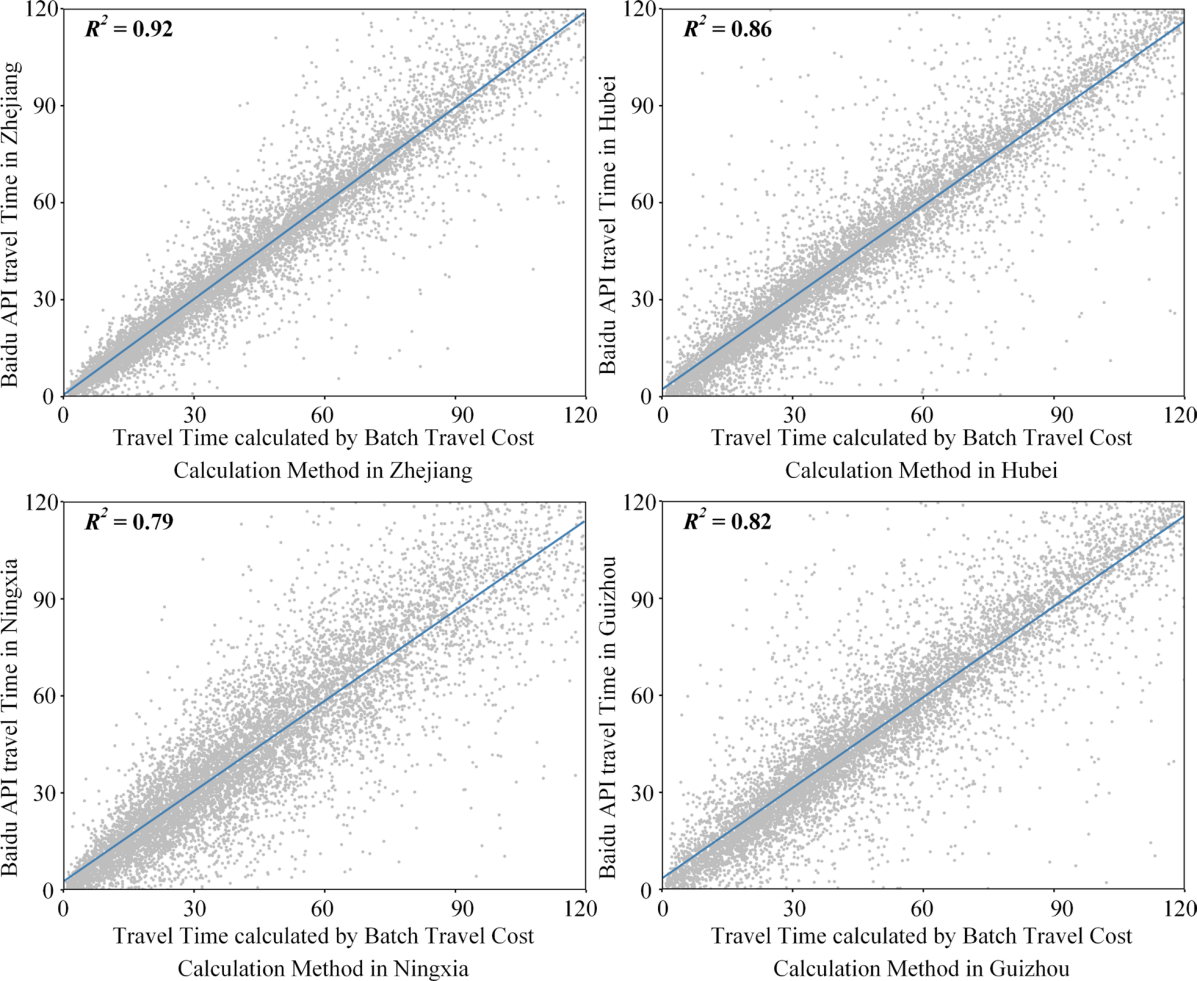
Comparison of travel time estimates from Baidu Map API and our model, using random OD samples selected from province levels

We then compared the travel time estimates produced by our method with those obtained from the Baidu Map API. A travel time threshold of 120 min was used to align the estimates. From each province, we randomly selected 10,000 OD pairs for comparison. As shown in Fig. 5, there was a strong positive correlation between our travel time estimates and those from the Baidu API, with R^2^ values ranging from 0.79 to 0.92 (all *p*-values < 10⁻^4^). Specifically, the coefficient of determination (R^2^) was 0.92 in Zhejiang, 0.86 in Hubei, 0.79 in Ningxia, and 0.82 in Guizhou.

In addition to the R^2^, we calculated the mean absolute percentage error (MAPE) to quantify the scale of differences between our travel time estimation and the Baidu API values. The MAPE was 15.24% in Zhejiang, 20.04% in Hubei, 24.96% in Ningxia, and 21.15% in Guizhou. It is important to note that the Baidu Map API incorporates real-time traffic conditions, which may be influenced by temporary factors such as congestion, road maintenance, and construction. These factors can introduce additional variability, potentially widening the observed differences. Therefore, these levels of error are considered acceptable for large-scale travel time estimation models. The high correlation and reasonable error margins in these provinces validate the robustness and applicability of our approach.

## Discussion

This paper introduced a comprehensive methodology for the batch calculation of travel costs to access healthcare services and demonstrates its application in the context of PHC facilities in Chinese mainland. This approach addresses several key limitations of existing PHC accessibility research and provides valuable insights into the spatial distribution of PHC access and its associated economic burden. The findings of this study have significant implications for health policies, resource allocation, and efforts to achieve universal health coverage.

### Progress and innovation of methodology

The methodology developed in this study represents a significant advancement in PHC accessibility research. Several methods have been employed in Western countries, including GIS-based models and time-based accessibility models, which focus on the accessibility of PHC services [30, 31]. Our study integrated various data sources, including high-resolution road network data, land use information, and detailed PHC facility locations, allowing for a more accurate representation of travel impedance than previous studies that relied on simpler distance measures or road network analyses [32–34]. The use of a modified version of Tobler’s bike function to account for the impact of terrain on travel speeds is particularly innovative and crucial for accurately modelling accessibility in topographically diverse countries such as China. Furthermore, the batch processing capabilities of our methodology enables the efficient analysis of vast geographical areas while maintaining high spatial resolution, addressing a key limitation of many existing approaches [23]. This scalability makes the proposed methodology particularly valuable for large-scale accessibility assessments and cross-regional comparisons. Our four-step approach, which encompasses friction surface map generation, PHC facility dataset construction, travel time calculation, and economic burden assessment, provides a comprehensive framework that can be adapted to various contexts and healthcare systems. A detailed comparison between our method and traditional methods is displayed in Appendix Table S1.

### Empirical applications in Chinese Mainland

This methodology revealed substantial disparities in PHC accessibility across Chinese mainland, with a clear east–west gradient in travel impedance. Approximately 88.70% of Chinese mainland’s population can access PHC facilities within an hour, but this figure masks significant regional and urban–rural variations. The stark contrast between the eastern coastal regions, which generally exhibit high accessibility, and the western inland areas, where travel times are considerably longer, underscores the persistent challenge of ensuring equitable PHC access in geographically diverse and economically disparate regions [35]. This pattern aligns with previous studies that have highlighted the urban–rural divide in PHC provisions in Chinese mainland [26]. Our high-resolution analysis provides a more nuanced understanding of these disparities, revealing pockets of poor accessibility even within well-served regions. This granular insight is crucial for identifying areas that require targeted interventions to improve PHC access. Similar challenges exist in other developing countries and research shows that in rural areas of India [36], half of the population cannot access outpatient and immunization services within one hour, with even worse conditions for delivery and inpatient care, where 42.2% and 47.2% of areas, respectively, are more than two hours away from service. A study conducted in sub-Saharan Africa [37] revealed that in 48 countries, fewer than 50% of the population in seven of these countries could reach a hospital within two hours of travel.The economic burden assessment conducted in this study offers a novel perspective on the hidden costs of PHC access disparities. The estimated total economic burden of 38.29 billion CNY annually associated with travel to PHC facilities in Chinese mainland is substantial and has not been previously quantified at this scale. This figure underscores the significant economic implications of PHC accessibility beyond the immediate health outcomes. The finding that the economic burden is not evenly distributed across Chinese mainland’s geographical divisions, with the southern, northern, and northwestern regions accounting for 62.91%, 21.72%, and 14.94% of the total burden respectively, highlights the complex relationship between economic development, population density, and PHC accessibility. Notably, the disproportionately high economic burden relative to GDP in the northwestern provinces suggests that improving PHC accessibility in these regions could yield significant economic benefits in addition to health improvements. These findings align with recent literature emphasizing the importance of considering the economic dimensions of PHC access in policy formulation [38, 39].

### Policy recommendations for improving PHC accessibility in Chinese mainland

The application of our methodology to China’s healthcare system provides valuable insights for health policy and planning, and supports several specific policy recommendations aimed at improving PHC accessibility across Chinese mainland. Regions with high travel impedance, especially western and remote areas, should receive prioritized investments in healthcare infrastructure, including establishing mobile clinics, enhancing transportation routes, and strategically locating new PHC facilities [40]. Targeted assistance from economically developed regions to remote areas with low accessibility is also effective. This concept aligns with recent calls for more comprehensive approaches to strengthen healthcare systems that consider both health and economic outcomes [41]. Expanding the use of telemedicine and digital health services is a key strategy [42] that can help provide timely consultations to residents in remote areas, reduce the need for long-distance travel, and improve healthcare accessibility for underserved populations. Digital health solutions can also reduce the economic burden associated with healthcare visits such as travel time and lost workdays. Based on multisource data and geospatial artificial intelligence (GeoAI), healthcare planning can be revolutionized by enabling more efficient and data-driven decision making [43]. By analyzing real-time data on healthcare facility locations, patient flow, and accessibility, GeoAI can support the optimization of healthcare resource allocation and help design more effective and responsive healthcare systems that adapt to the specific needs of different regions.

### Contributions to global health

In addition to its application in Chinese mainland, the methodology developed in this study offers a versatile assessment method for PHC services worldwide as it was designed with high parametric flexibility. By leveraging openly accessible datasets such as road networks, surface features, and national statistics (e.g., GDP and population), our method allows for the analysis of PHC travel times across a wide range of geographical contexts. This adaptability ensures that the core logic for calculating travel time is not only applicable to Chinese mainland but can also be extended to other countries or regions with similar healthcare accessibility challenges. By adjusting key parameters such as GDP or population density, our framework can support PHC accessibility assessments under various global scenarios, making it highly relevant for countries facing similar barriers to healthcare access, particularly low- and middle-income countries.

Furthermore, this study yielded methodological innovations that address existing gaps in global health research, particularly in the areas of spatial analysis and health economics. The computational methods developed were designed to overcome challenges related to data accessibility and the universality of computational logic, allowing for an efficient and scalable analysis of healthcare accessibility. Notably, patented core methods demonstrate significant practical value, highlighting their potential for optimization and broader dissemination. This approach can be further enhanced in the future by integrating additional data sources such as dynamic traffic data and regional health information to improve its adaptability and applicability in diverse settings.

Additionally, by quantifying PHC accessibility in terms of travel time and economic burden, our framework offers critical insights into global health policies, aligning closely with global health governance objectives, particularly SDG 3.8, which aims for universal health coverage. Our methodology provides policymakers with a robust data-driven framework for optimizing resource allocation in PHC systems. The findings of this study can help guide efforts to reduce disparities in healthcare access and ensure that healthcare resources are allocated more efficiently and equitably. Ultimately, this methodology holds significant promise for informing policies that are designed to enhance healthcare equity and access.

### Limitations

Despite its strengths, this study had several limitations that should be addressed in future research. First, although our methodology accounts for various factors affecting travel speed, it does not capture all potential barriers to PHC access, such as cultural factors, personal preferences, or the quality of care provided at different facilities. Incorporating these factors into future accessibility models could provide a more comprehensive understanding of PHC access challenges. Second, our economic burden assessment, which is more comprehensive that of many previous studies, still relies on several assumptions and generalizations. Future studies could refine this assessment by incorporating more detailed data on PHC utilization patterns, transportation costs, and productivity losses. Finally, although our methodology was designed to adapt to different contexts, its application in other countries may require adjustments to account for local data availability and specific PHC system characteristics. Testing and refining this methodology in diverse global contexts are important areas for future research.

## Conclusions

The findings of this study highlight notable disparities in access to PHC across Chinese mainland and quantify the associated economic burdens. The developed methodology offers a powerful and robust tool for researchers and policymakers to conduct large-scale, high-resolution accessibility analyses, which can offer valuable insights to guide evidence-based interventions for enhancing PHC equity. As countries worldwide strive to achieve universal health coverage, approaches like the one presented in this study will be crucial for monitoring progress, identifying challenges, and guiding policy decisions. Future research should focus on refining and expanding this methodology to incorporate additional factors affecting PHC access and applying it to diverse global contexts.

## Supplementary Information

Additional file 1.

## Data Availability

The datasets generated during and analyzed during the current study are available from the corresponding author on reasonable request.
