# Supplementary material for: Beyond distance: integrating economic burden into large-scale primary healthcare accessibility analysis
Source: Glob Health Res Policy. 2025 Oct 27;10:53. doi: 10.1186/s41256-025-00451-9 (PMC12557955; doi:10.1186/s41256-025-00451-9)
Supplement: Supplementary file 1 — Additional file 1. [file 41256_2025_451_MOESM1_ESM.docx]

**Supplementary Appendix**

**Beyond Distance: Integrating Economic Burden in Large-Scale Healthcare Accessibility Analysis**

**Fig. S1. Travel time of community/township healthcare centers and clinics in China**


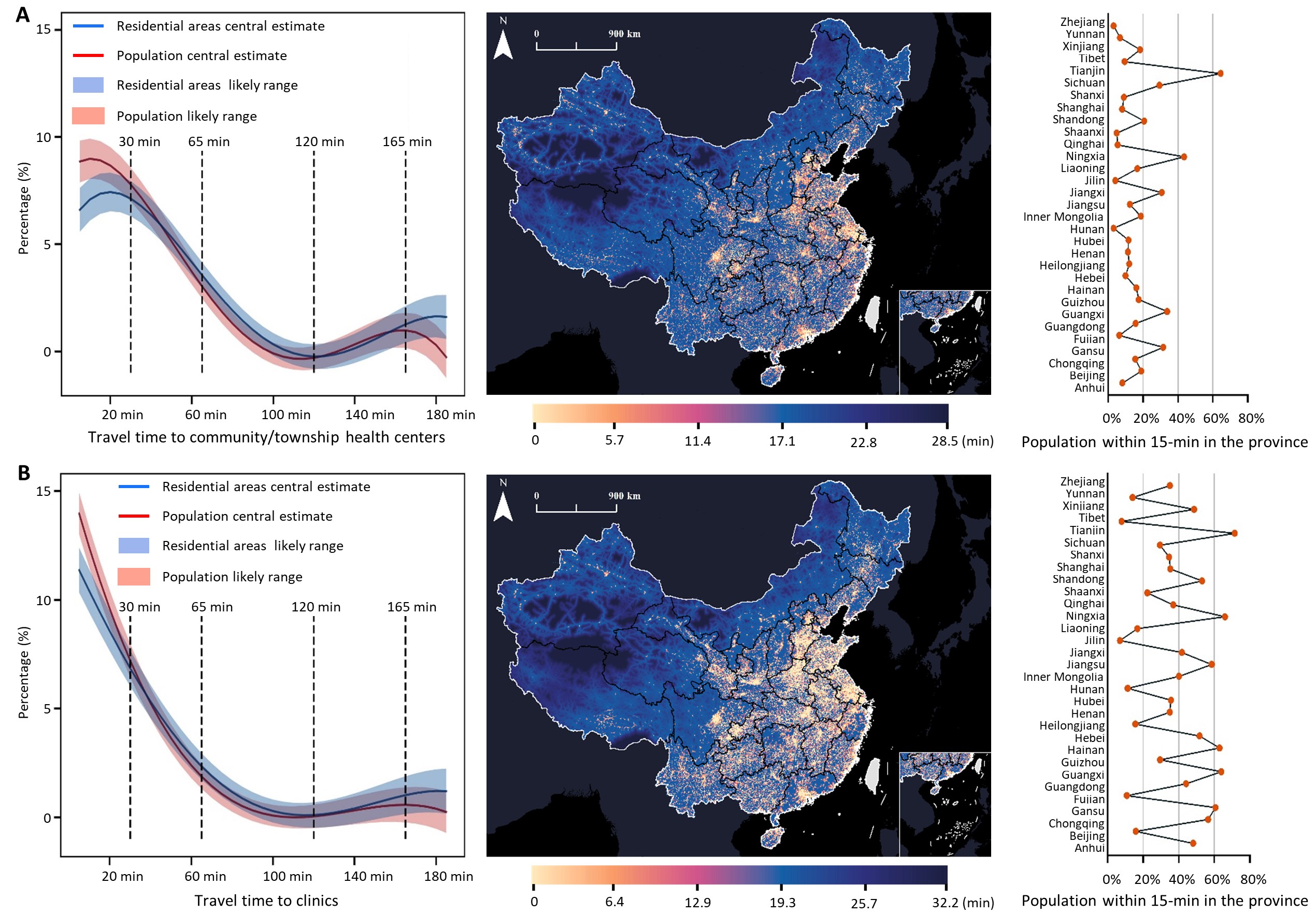


**Note:** Here, we further reveal the regional differences in travel time of primary healthcare institutions in China, and show the population characteristics covered by the two types of primary healthcare service institutions under different time thresholds (Left), spatial distribution (Middle), and the proportion of people in different provinces who can assess healthcare services within 15 minutes (Right). For primary healthcare institutions established in the same level of administrative units, the eastern region is also better than the western region.

Table. S1. The advantages and disadvantages between our method and traditional methods in travel time calculation

| **Methods** | **Advantages** | **Disadvantages** |
| --- | --- | --- |
| Straight-Line Distance Method | Simple and easy to implement | Significant deviation from actual travel time;  Unsuitable for complex transportation networks |
| Survey-Based Method | Based on individuals’ actual travel behaviors | Accuracy may be low due to personal subjectivity;  Collection can be costly |
| Real-time Navigation Method | High accuracy and reflects dynamic traffic conditions | The calculative cost is high;  Difficult to batch calculation;  Limited applicability in remote areas |
| **Our method** | Batch calculating the travel time;  Integrating multiple geospatial datasets;  Related data is easy to access | Less accurate than real-time navigation method |
